# Supplementary figures and images for: Comparative cytogenetics of three Zoraptera species as a basis for understanding chromosomal evolution in Polyneoptera insects
Source: PeerJ. 2024 Oct 10;12:e18051. doi: 10.7717/peerj.18051 (PMC11471171; doi:10.7717/peerj.18051)

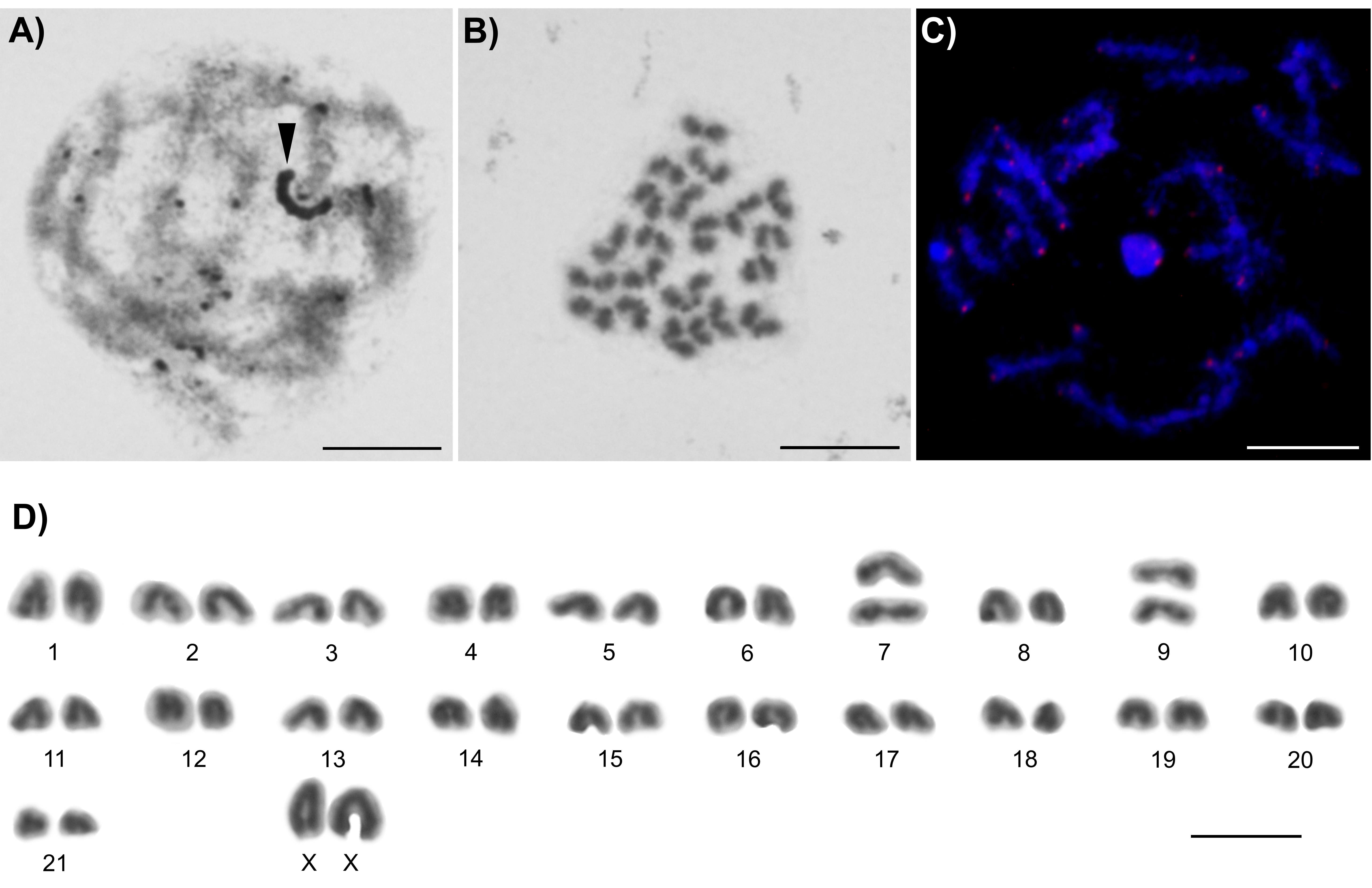

Supplement: Supplemental Information 1 — (A, B, D) and with DAPI (blue) (C). The arrowheads indicate sex chromosomes. The scale bars correspond to 10 μm. (A) Male Brazilozoros huxleyi, pachytene. (B) Male Brazilozoros kukalovae ( n = 21), one sister metaphase II without an X chromosome. (C) Male Latinozoros cacaoensis, pachytene after FISH with the (TTAGGG)8 probe (red signals). (D) Female Brazilozoros kukalovae ( 2n = 44); karyogram based on mitotic metaphase. [file peerj-12-18051-s001.jpg]
