# Supplementary material for: Comparative cytogenetics of three Zoraptera species as a basis for understanding chromosomal evolution in Polyneoptera insects
Source: PeerJ. 2024 Oct 10;12:e18051. doi: 10.7717/peerj.18051 (PMC11471171; doi:10.7717/peerj.18051)
Supplement: Supplemental Information 2 [file peerj-12-18051-s002.docx]

**Sequences of probes used for FISH.**

**L. cacaonsis H3:**

AAGGAAATCCACTGGAGGTAAAGCACCTCGCAAACAACTTGCTACTAAAGCTGCCCGTAAAAGTGCCCCGGCTACCGGAGGAGTCAAGAAACCCCATAGGTACAGGCCTGGCACTGTCGCATTGAGAGAAATTCGGCGTTACCAGAAGAGCACCGAACTCTTGATTCGCAAACTTCCATTCCAAAGATTAGTTCGTGAGATTGCTCAGGATTTTAAGACTGACTTACGATTTCAGAGCTCCGCAGTCATGGCTTTACAGGAGGCAAGTGAAGCTTACCTCGTAGGATTGTTTGAAGACACCAACTTGTGTGCAATTCATGCCAAGAGG

**B. huxleyi H3:**

GCAAGAAAATCAACTGGAGGCAAGGCCCCTCGCAAACAGCTCGCTACAAAAGCTGCTCGCAAAAGTGCTCCAGCCACCGGAGGTGTAAAGAAGCCACATAGATACAGGCCAGGAACTGTAGCTCTGAGAGAAATCCGTCGTTATCAAAAGAGCACTGAGCTTCTCATTCGCAAACTTCCCTTCCAAAGGTTGGTTCGTGAAATCGCCCAGGATTTCAAAACAGATTTGAGATTCCAGAGCTCGGCTGTTATGGCTTTACAGGAAGCAAGCGAAGCTTACC

**B. huxleyi 18S:**

ATGGCTCAGTAAATCAGTTACGGTTCATTTGATGAATTGTAAAAAGACGTGGATAACTGTGGCAATTCCAGAGCTAATACATGCCATGAAGTCCCGACCTTGCGGAAGGGACGCTTTTATTAGAATACAAAACCAGTCCGAGGTTTGGCAGAAATGTCTTATCGAAACTCCCAGTGATGAATCTGAGTAACATTGAACAGATCGCATGGCCCTTGAGCAAGGCGACGCATCATTCAAACGCCTGCTCTATCAACTTTGATGGCCGATTATACGCCTACCATGGTGTTGACGGGTGACGGGGAATCGGGGTTCGATTCCGGAGAGGGAGCCTGAGAAACTGCTACCACATCCAAGGAAGGCAGCAGGCGCGCAAATTACCCACTCCCGGCACGGGGAGGTAGTGACGAAGTATAACGATACGGGACTCCCATCCGAGGCCCCGTGATCGGAATGAGACCGGTTCAGAGAACTGGTCCGAGTATCCATTGGAGGGCAAGCCTGGTGCCAGCAGCCGCGGTAATTCCAGCTCCAATAGCATATACTAATGTTGTTGCGGTTAAAAAGCTCGTAGTCAGAGTTGTGTCCCGCGCTGCCGGTCCACAGACTGTCTGTGCCGACTGCGCACGTCGCGAGGACGTCCTGGCCGGTAGCGGTGTAGCCGAAGTTCCGACGACGTACAGGTTCGCCCTGTGCCCGGCGTTACCTAGGTGAACCGCAATTTAGGTCCCGACCTGGTGCTCTTAATCGAGTGCCTAGGAGGGCCGGCACTCTTACTTTGAACAAACTCGAGTGCTCAAAGCTGGCCTTCTGGCCTAGACGCTGTGTGCATGGAATAATGGAATAGGACTTCGGTTCTATTTGGTTGGTTCTAAGAACCGAAGTAATGATCAAAAGGAGCAGGCGGGGGCATTCGTACTGCGACGTTAGAGGTGAAATTCTTAGATCGTCGCAAGACGGACAAAAGCGAAAGCATTTGCCAAGGATGTTTCCACTGATCAAGAGCGAAAGTTGGACGATCGAAGGCGATCAGATACCGCCCTAGTTCTAACTATAAATGATGCCAGCCAGCTATGCACCGCAGTCGCCCTTGACTCGGAGCGAAGCTTCCGGGAAACCAGAGCTTTCAGGTTCCGGGGGAAGTATGGTTGCAAAGCTGAAACTTAAAGGAATTGACGGAAGGGCACCACCAGGAGTGGAGCTTGCGGCTTAATTTGACTCAACACGGCAAAATTTATCAAGGCAGGACAAACGTAGGATTGACAGATTGAAGATCTCTCTTGATTGTTTGGGTGGTGGTGCATGGCCGTTCTTAGTTCGTGGACTGGTCCGTCTGGTTAATTCCGATAACGAACGAGACTCTAACCTGCTAAATAGTCCAAATTGAGATCCCTTCGGGGCTATCTGAGAGAAATCCTTCTTAGAGGAAACAGCGGCCTCGAGTCGCATGATATAGAGCAATAACAGGTCTGGTATGCCCTTAGATGTCTTGGGCCGCACGCGTGCTACAATGAAGAAAGCAGCGTGAAAAATCCTAGGCCGAGAGGTCCGGGTAATCAGCTCAAACTTTTTCATGCAGGGGATAGGGGCTTGCAATTGACCCCTTGAACAAGGAATTCCCAGTAGGCGCGAGTTATGAGCTCGTGTCGATTGAGTCCCTGCCCTTTGTCCACACCGCCCGTCGCTACTTCCGATCGAATGGGGTAGTGAGGTCTCCGGACCGATGCTATAGGTCCAAAAAAACCTTAGCTTTGGGAGGCTGACCAAA
